# Genome-wide DNA methylation comparison between live human brain and peripheral tissues within individuals

**DOI:** 10.1038/s41398-019-0376-y

**Published:** 2019-01-31

**Authors:** Patricia R. Braun, Shizhong Han, Benjamin Hing, Yasunori Nagahama, Lindsey N. Gaul, Jonathan T. Heinzman, Andrew J. Grossbach, Liesl Close, Brian J. Dlouhy, Matthew A. Howard, Hiroto Kawasaki, James B. Potash, Gen Shinozaki

**Affiliations:** 1 Department of Psychiatry, University of Iowa Carver College of Medicine, Iowa City, IA 52246 USA; 2 Interdisciplinary Graduate Program in Genetics, University of Iowa, Iowa City, IA 52246 USA; 3 Department of Psychiatry and Behavioral Science, Johns Hopkins University School of Medicine, Baltimore, MD 21287 USA; 4 Department of Neurosurgery, University of Iowa Carver College of Medicine, Iowa City, IA 52246 USA; 5 Department of Neurological Surgery, Ohio State University, Columbus, OH 43203 USA; 6 Iowa Neuroscience Institute, University of Iowa Carver College of Medicine, Iowa City, IA 52246 USA; 7 Interdisciplinary Graduate Program for Neuroscience, University of Iowa Carver College of Medicine, Iowa City, IA 52246 USA

## Abstract

Differential DNA methylation in the brain is associated with many psychiatric diseases, but access to brain tissues is essentially limited to postmortem samples. The use of surrogate tissues has become common in identifying methylation changes associated with psychiatric disease. In this study, we determined the extent to which peripheral tissues can be used as surrogates for DNA methylation in the brain. Blood, saliva, buccal, and live brain tissue samples from 27 patients with medically intractable epilepsy undergoing brain resection were collected (age range 5–61 years). Genome-wide methylation was assessed with the Infinium HumanMethylation450 (*n* = 12) and HumanMethylationEPIC BeadChip arrays (*n* = 21). For the EPIC methylation data averaged for each CpG across subjects, the saliva–brain correlation (*r* = 0.90) was higher than that for blood–brain (r = 0.86) and buccal–brain (*r* = 0.85) comparisons. However, within individual CpGs, blood had the highest proportion of CpGs correlated to brain at nominally significant levels (20.8%), as compared to buccal tissue (17.4%) and saliva (15.1%). For each CpG and each gene, levels of brain-peripheral tissue correlation varied widely. This indicates that to determine the most useful surrogate tissue for representing brain DNA methylation, the patterns specific to the genomic region of interest must be considered. To assist in that objective, we have developed a website, IMAGE-CpG, that allows researchers to interrogate DNA methylation levels and degree of cross-tissue correlation in user-defined locations across the genome.

## Introduction

Using human postmortem brain tissues, epigenetic studies among psychiatric populations have found differential DNA methylation (DNAm) changes in both candidate gene studies and genome-wide analyses, including in schizophrenia^1^, bipolar disorder^2^, and major depressive disorder (MDD)^3^, but access to brain tissue for psychiatric disorders is limited by a small number of postmortem brain samples. Additionally, considerations remain regarding the stability and the biological implications of measurements done on postmortem tissues, as the levels of global DNAm have been shown to change in relation to postmortem interval^4,5^. Consequently, psychiatric epigenetic studies with peripheral tissues, such as blood or saliva samples, have become common^6–12^. However, the extent to which epigenetic marks from peripheral tissues can be used to represent or predict those of brain tissues from live humans at the same moment in time is unknown.

Several studies have begun to address the degree to which DNAm of peripheral tissues correspond to that of the brain. Earlier studies approached this using an across-individual method. Horvath et al.^13^ found high levels of correlation (*r* = 0.85–0.91) with genome-wide methylation data from a large number public datasets by taking the means of methylation values across samples between blood, and four different brain regions of different individuals using probes that overlapped between the Illumina 27K and 450K arrays. A study by Davies et al.^14^ found with MeDIP-seq data from three individuals that CpGs in promoter regions with high CG content had higher correlations between blood and the cortex and cerebellum (*r* = 0.82 and 0.77, respectively) than CpGs in promoters with low CG content (*r* = 0.31 and 0.40, respectively). A comparison between DNAm in saliva and blood from 64 living individuals and DNAm in separate postmortem brain samples found that saliva may be more informative than blood as a surrogate for brain DNAm^15^.

However, more recent studies have instead interrogated the degree of correlation within-individuals. These include three studies using whole blood samples that have reported that only a small percentage of CpGs are both variable in DNAm and significantly correlated with brain DNAm. A large study with 80 matched whole blood and postmortem brain tissues from the same individuals found that among the CpGs on the Illumina 450K array noted to show interindividual DNAm variability in blood-derived DNA, only 1.4% were found to be strongly predictive of prefrontal cortex DNAm variation^16^. In a comparison between 12 live brain tissues from epilepsy patients with whole blood samples from the same group, Walton et al.^17^ found only a small portion (7.9%) of variable CpGs on the Illumina 450K array showed significant correlation in DNAm between tissues. Also using the Illumina 450K array, DNAm from paired samples of blood and three postmortem brain regions from 16 individuals was used to develop a tool called BECon that investigates how informative DNAm from the blood is for brain DNAm, for which they found that ~9.7% of the CpGs were informative^18^.

While these studies have provided valuable information about the usefulness of surrogate tissues, some important questions remain, including the relationship of DNAm in live human brain tissue and DNAm: (1) across different types of peripheral tissues; (2) within the same individuals; and (3) within tissues collected at the same point in time. To address these vital issues, we collected brain tissues resected during neurosurgical intervention for subjects with medically intractable epilepsy, and in conjunction, obtained saliva, blood, and buccal tissue samples to compare genome-wide DNAm using the Illumina 450K and EPIC array platforms.

## Materials and methods

### Participants and sample collection

Twenty-seven subjects with medically intractable epilepsy undergoing neurosurgery were recruited for this study between March 2014 and April 2017 at the University of Iowa Hospitals and Clinics. The cohort included a wide age range (5–61 years) with an average age of 32 ± 15.8. This study was approved by the University of Iowa’s Human Subjects Research Institution Review Board. Written informed consent was obtained. Pathology reports of resected brain tissues showed samples with a range of pathologies, including gliosis, sclerosis, and focal cortical dysplasia (Supplemental Table 1). The exclusion criterion for subjects was having a tumor by pathological report. Whole blood samples were collected in EDTA tubes, saliva with the Oragene DISCOVER™ kit (DNA Genotek Inc., OGR-500), and buccal tissue with swabs (Puritan, 25–1506 1PF TT MC). Resected brain tissue samples were immediately stored and transported on dry ice and a portion of each brain region was sent to pathology. All samples were stored at −80 °C. Typically, blood samples were taken at the end of surgery in the operating room, and saliva and buccal swabs were collected at 2 days after the operation; however, for eight subjects, buccal samples were not obtained until the patients returned for follow-up appointments at the neurosurgery clinic. For these subjects, additional saliva samples were taken at the same time to be analyzed in conjunction with the retrospective buccal samples. For each sample, date of acquisition in relation to the date of surgery was recorded (Supplemental Table 1).

### Methylome assays

Genomic DNA was extracted from whole blood, buccal, saliva, and brain tissues with the MasterPureTM DNA extraction kit (Epicenter, MCD85201) following the respective protocols for each tissue type. DNA quality was assessed with NanoDrop spectrometry and quantified with the Qubit™ dsDNA Broad Range Assay Kit (ThermoFisher Scientific, Q32850).

For each sample, 500 ng of DNA was bisulfite converted with the EZ DNA Methylation™ Kit (Zymo Research, D5002). Genome-wide DNAm was assessed using the Infinium HumanMethylation450 BeadChip Kit (Illumina, WG-314-1003) array on 12 subjects, initially, for brain, blood, and saliva samples. The Infinium HumanMethylationEPIC BeadChip™ Kit (Illumina, WG-317-1002) was used to analyze 21 subjects with brain, blood, saliva, and buccal samples, with six subjects overlapping between the two datasets (Supplemental Table 1). Samples were grouped by individuals and randomized onto the chips. The arrays were scanned with the Illumina iScan platform. High degrees of correlation were seen using the beta values between samples run on both the Illumina 450K and EPIC arrays and with probes that overlapped both platforms (*N* = 15, *r*^*2*^ > 0.98).

The methylation data were processed with the R packages Minfi and RnBeads^19–21^. Background correction was performed with the Noob method in Minfi^20,22^. Using RnBeads, probes were filtered out if they: (1) overlapped within 5 bp of a SNP (EPIC: 21,358 probes), (2) had a detection *P-*value > 0.01 or were considered unreliable measures based on RnBeads’s greedy-cut algorithm (EPIC: 18,864 probes), or (3) were context-specific sites (EPIC: 2,873 probes). Probes excluded with overlapping SNPs were assigned by RnBeads using the version of dbSNP derived from Genome Reference Consortium Human Build 37 patch release 10 (GRCh37.p10)^21^. After filtering, 822,996 probes remained for the EPIC dataset. Samples were normalized with beta mixture quantile dilation (BMIQ)^23^. Samples from the same individual were verified by generating a heatmap cluster of the 65 SNP probes on the array.

### Statistical analysis

All statistical analyses were performed in R^24^. Two approaches to cross-tissue correlation were used. First, overall levels of DNAm correlation were calculated from the average methylation across subjects for each tissue using Pearson’s correlation. For the overall correlation, all 822,996 CpGs were used in the calculation, but for the genomic, functional, and gene-based correlations, the subset of CpGs based on their location were used to determine the degree of correlation based on the tissue averages. The R package cocor was used to compare two correlations based on dependent groups^25^. Second, a within-subjects approach was used. For each individual CpG, a correlation coefficient and its significance level were calculated with Spearman’s test because of the small sample size. For comparisons between tissues, the average of the individual CpG correlation coefficients were taken from all CpGs in the genomic, functional, or gene-based groups. Variable CpGs were classified as by Hannon et al.^14,16^ This approach involved excluding DNAm values in the upper and lower 10th percentile for each CpG, then classifying as variable those CpGs where the remaining range differed by at least 5%.

### CpG classification

Genomic regions were classified using the annotation file provided by Illumina with genic regions based on the UCSC annotation and functional regions as determined by the ENCODE project.

### Brain-expressed genes

The Expression Atlas database was used to identify brain-expressed genes (https://www.ebi.ac.uk/gxa/home). ENCODE RNA-seq data were provided by the ENCODE Consortium^26^, and as defined by the Atlas, medium expression level included transcripts above 11 fragments per kb of transcript per million mapped reads.

### mQTL classification

A list of CpGs associated with SNPs was derived from a database of DNAm quantitative trait loci (mQTL; http://www.mqtldb.org/)^27^. Gaunt et al.’s original *p*-value cutoff of *p* < 1 × 10^−14^ was used, resulting in 27,448 CpGs under genetic influence that overlapped with our study. For comparison, the remaining CpGs that overlapped with the 450 K dataset were used and classified as non-mQTL (*n* = 400,932).

### Fluorescence-activated cell sorting

Fluorescence-activated cell sorting was performed on brain samples from all subjects. Briefly, brains were homogenized using a 15-ml tissue grinder, potter–ELV (Wheaton) in nuclei extraction buffer (320 mM sucrose, 5 mM CaCl_2_, 3 mM Mg(AC)_2_, 0.1 mM ETDA, 10 mM Tris-HCl, 1 mM Dithiothreitol, 0.1% Triton X, 1 tablet of cOmplete™ mini protease cocktail inhibitor (Roche, cat. #11836153001)). Homogenates were filtered using 40 µM cell strainer and transfer into ultracentrifuge tubes (Beckman Coulter, cat. #344059). Sucrose cushion (1800mM sucrose, 3 mM Mg(AC)_2_, 1 mM dithiothreitol, 10 mM Tris-HCl) was layered under the homogenates until the homogenates were at the brim of the tube. Ultracentrifuge tubes were contained in SW45-Ti swing bucket rotor (Beckman Coulter) and centrifuged using L8–70M ultracentrifuge (Beckman Coulter) at 25,000 rpm for 2.5 h at 4 °C. Supernatant was removed and the pellet was introduced to chilled phosphate buffered saline and incubated on ice for 20 min prior to resuspension by gentle pipetting. Suspended nuclei were exposed to blocking solution (0.5% bovine serum albumin, 10% normal goat serum, phosphate buffered saline, pH 7,4) in a 10:1 ratio and incubated at 4 °C for 1 h. Following this, nuclei were stained using either Alexa Fluor®488 conjugated anti-NeuN antibody (Millipore, cat. #MAB377X) or its isotype control (Millipore, cat. #16–240) in 1:1000 or 1:500, respectively to a final concentration of 1 µg/ml for both antibodies. Nuclei-antibody mix was incubated overnight at 4 °C prior to FACS. Nuclei were sorted at the University of Iowa flow cytometry facility using the Becton Dickinson FACS fusion (Becton Dickinson Biosciences). NeuN− nuclei were identified using the Alexa Fluor®488 conjugated isotype control and autofluorescence using the PerCP-Cy5-5 channel. NeuN+ nuclei were identified from an increase in Alexa Fluor®488 signal intensity above that of the isotype control and limited autofluorescence detected from the PerCP-Cy5-5 channel (Supplemental Figure 1A). To validate the purity of NeuN+ and NeuN−nuclei enriched by FACS, bisulfite-pyrosequencing was performed on the glial fibrillary acidic protein (GFAP) promoter (Supplemental Table 2) from hippocampal tissues of two individuals. Consistent with GFAP expression in glial cells but not in neurons^28^, GFAP DNAm levels were observed to be high in NeuN+ but low in NeuN–nuclei (Supplemental Figure 1G).

### Cellular compostion adjustment

To compare the correlations of the unadjusted and adjusted datasets, brain and blood samples were adjusted with the method proposed by Jones et al.^29^ Cell-type proportions were estimated and used to identify the amount of variation in methylation values explained and adjust them accordingly.

## Results

Genome-wide DNAm levels were assessed from brain and peripheral tissues of 27 subjects. Preliminary analyses were performed with the Illumina 450K Array on 12 individuals with brain, saliva, and blood samples. The Illumina EPIC Array was then performed on brain, saliva, blood, and buccal samples from 21 subjects, with six subjects overlapping between the two arrays. Samples were obtained from patients undergoing neurosurgical resection for medically intractable epilepsy, and subject characteristics can be found in Supplemental Table 1. Unless otherwise noted, data presented are from the EPIC array.

**Fig. 1:**
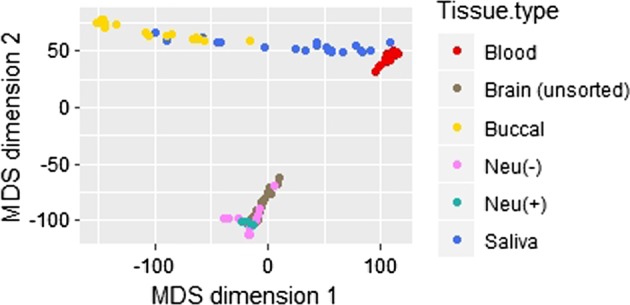
Multidimensional scaling (MDS) plot of genome-wide DNAm levels from brain, saliva, blood, buccal samples (*n* = 21) and FAC-sorted brain samples (neu(+) *n* = 5, neu(−) *n* = 12) analyzed on the Illumina EPIC array

To determine the degree of similarity in genome-wide DNAm between brain and peripheral tissues, a multidimensional scaling (MDS) plot was constructed with all the samples for both datasets. In the initial 450K analysis, two brain samples consistent with oligodendroglioma clustered separately from the other brain tissues and those subjects were thus removed from further analyses (Supplemental Figure 2), leaving only the patients with epilepsy who are the focus of this report. Similar patterns in the MDS plots were seen for each platform. The MDS plot with the EPIC dataset revealed the brain samples clustering separately from all peripheral tissues with the peripheral tissues clustered as expected based on their cellular compositions (Fig. 1). That is, the saliva samples, being a mixture of ~70% leukocytes and 30% epithelial cells^30^, clustered between the blood and buccal samples. Although predominantly epithelial cells, the obtained buccal samples also likely contain saliva tissue, thus leukocytes as well. As such, the buccal samples clustered together but some samples overlapped with the saliva samples, suggesting a variable level of leukocytes in the buccal samples. Similarly, the saliva samples were distinctly clustered, but were spread over a wide range between the buccal and blood samples, possibly indicating variable levels of epithelial cells and leukocytes within them (Fig. 1).

We assessed cross-tissue correlations in DNAm in two different ways. The first was an approach that averaged DNAm across subjects, while the second focused on a within-subject analysis of individual CpGs. The former represents a global picture of all CpGs represented on the array. For each tissue, the methylation values are averaged across all subjects, and then the correlation of all CpGs between two tissues is calculated. The latter is an individual CpG-focused approach. For each CpG, the correlation is calculated separately using distinct data points from each subject. This individual CpG-focused approach allows us to determine which CpGs demonstrate variability, a feature that enhances the likelihood of disease-relevance. Thus, for the within-subject analysis, we focused on variable CpGs as we examined cross-tissue correlation.

### Across-subject correlations

Overall levels of genome-wide DNAm correlation were calculated from the average methylation across all CpGs between each peripheral tissue and brain using Pearson’s correlation (Table 1). Among comparisons between peripheral tissues, blood, and saliva showed the highest degree of correlation for DNAm (*r* = 0.97), followed by saliva and buccal tissue (*r* = 0.93), then blood and buccal tissue (*r* = 0.81). When peripheral tissues were compared to brain, relatively high levels of correlation were observed (blood–brain *r* = 0.86, buccal–brain *r* = 0.85, saliva–brain *r* = 0.90). Genome-wide DNAm of saliva was significantly more correlated with DNAm in brain than was either blood (*p* < 2.2 × 10^−16^) or buccal tissue (*p* < 2.2 × 10^−16^).

**Table 1:** Pearson’s correlations were calculated between each tissue from the tissues’ average methylation for all CpGs

Tissues compared	Overall correlation in DNAm (rho)	Correlation of variable CpGs (rho)
Brain vs. blood	0.86	0.63
Brain vs. buccal	0.85	0.59
Brain vs. saliva	0.90	0.75
Saliva vs. blood	0.97	0.91
Saliva vs. buccal	0.93	0.73
Blood vs. buccal	0.81	0.45
Neuronal negative vs. blood	0.85	0.61
Neuronal negative vs. buccal	0.84	0.58
Neuronal negative vs. saliva	0.89	0.71
Neuronal negative vs. brain	0.99	0.98
Neuronal positive vs. blood	0.79	0.50
Neuronal positive vs. buccal	0.80	0.53
Neuronal positive vs. saliva	0.82	0.59
Neuronal positive vs. brain	0.92	0.75
Neuronal positive vs. neuronal negative	0.92	0.76

Among the four subjects with 450K data that had three brain regions (amygdala, hippocampus, and temporal cortex) analyzed, saliva DNAm was more highly correlated than blood DNAm to each region, in a pattern similar to the overall peripheral tissue to average brain comparisons (amygdala–blood *r* = 0.92 vs. amygdala–saliva *r* = 0.93; hippocampus–blood *r* = 0.91 vs. hippocampus–saliva *r* = 0.93; temporal cortex–blood *r* = 0.90 vs. temporal cortex–saliva *r* = 0.92).

### Within-subject correlations at each CpG

**Fig. 2:**
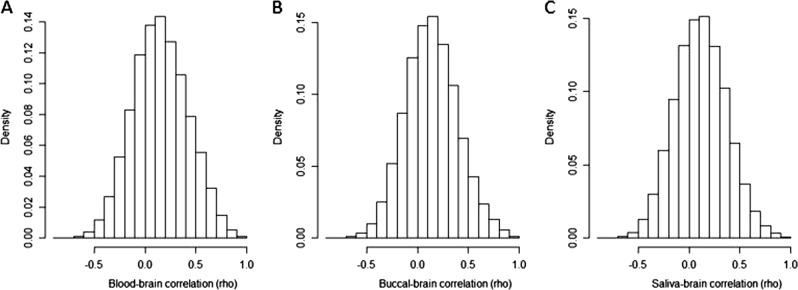
Histograms of the distribution of correlations (*r*) of the individual 822,996 CpGs covered on the EPIC array between the brain and **a** blood, **b** buccal, and **c** saliva tissues (*n* = 21)

A within-subject comparison at the individual CpG level revealed only a fraction of CpGs to have significant correlations between the peripheral tissues and brain. Of the 822,996 CpGs, 20.8% were correlated at a nominal level of significance in blood (*p* < 0.05), 17.4% in buccal tissues, and 15.1% in saliva, each with a *r* > 0.38. Moderately strong correlations (*r* > 0.5) was seen in 10.9% of the CpGs in blood, 8.5% in buccal, and 6.7% in saliva. The mean and median correlations for blood were *r* = 0.15 and 0.14, respectively, for buccal *r* = 0.14 and 0.13, and for saliva *r* = 0.12 and 0.11. Figure 2 shows the distribution of correlations for each peripheral tissue’s correlation to the brain across all CpGs. Of the variable CpGs for each peripheral tissue (blood = 352,824, buccal = 427,048, and saliva = 485,203), with variability calculated as described by Hannon et al.^16^, 26.6% of the variable CpGs in blood, 20.4% in buccal, and 17.6% in saliva were nominally correlated. Additionally, 15.7% of the variable CpGs in blood, 11.6% in buccal, and 8.8% in saliva were moderately correlated (*r* > 0.5). These within-subject comparisons contrast with the correlations that were seen across the average methylation values for all CpGs, where saliva had the greatest degree of correlation. A portion of the individual CpGs survived Bonferroni correction for multiple testing (*p* < 6.1 × 10^−8^). This included 2,357 CpGs for blood, 2,367 CpGs for buccal, and 1,498 CpGs for saliva, with corresponding rho values greater than 0.83 (Supplemental Table 3).

### Correlations by subject

**Fig. 3:**
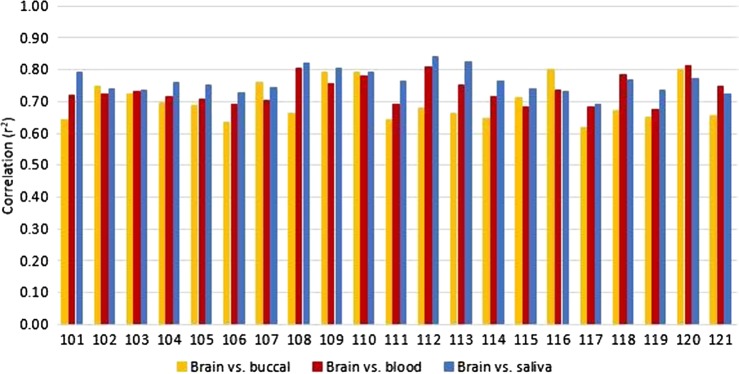
Degree of DNAm correlation of all CpG probes between each peripheral tissue and brain for all subjects

Next, we assessed the consistency of the correlations within individuals. Among the 21 subjects, the saliva–brain correlation was greater than the blood–brain correlation for all but four individuals. The buccal–brain correlation was lowest in each individual except for five individuals for whom the buccal–brain correlations were higher than blood–brain, and five individuals for whom the buccal–brain correlation was higher than saliva–brain (Fig. 3). For the subjects with the higher buccal–brain correlations, the buccal samples also had higher buccal–blood correlations. On the MDS plot (Fig. 1), the buccal samples with the highest buccal–brain correlation clustered closest to the saliva and blood samples. The relationship between the correlations of buccal–blood and buccal–brain were significant, in that as the buccal–brain correlation increased within subjects, their buccal–blood correlations also increased (*r*^2^ = 0.88, *F* = 142.8, *p* = 2.8 × 10^−10^); this was not true between the buccal–brain and buccal–saliva correlations (*r*^2^ = 0.01, *F* = 0.21, *p* = 0.65; Supplemental Figure 3). Additionally, the MDS distances between the peripheral samples and the brain samples from the same individual were calculated. As the MDS distance between the peripheral tissues and brain decreased, the level of correlation increased (Supplemental Figure 4). This suggests that the buccal samples are: (1) not a near pure population of epithelial cells and likely have varying portions of leukocytes, although in a lower proportion than saliva, and (2) the highest peripheral-brain correlation comes from a mixture of cell compositions as evidenced by the saliva–brain correlations being highest in most subjects, and by the buccal–brain correlations being highest in buccal samples that most resembled saliva on the MDS plot.

### Impact of genomic and functional regions

**Fig. 4:**
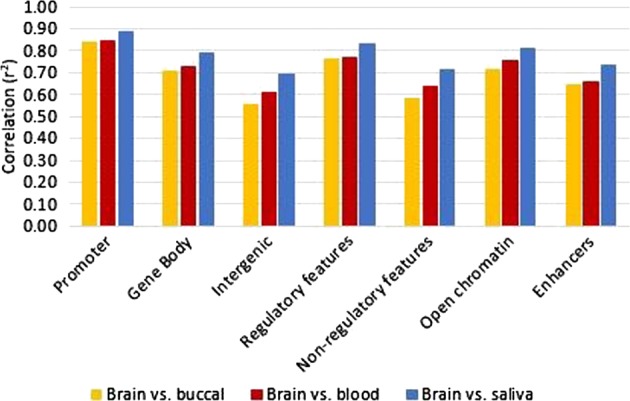
DNAm correlation comparisons of CpGs located in different genomic and functional contexts taken from the average DNAm for each tissue

Analysis of DNAm correlation between peripheral tissues and brain among CpGs in different genomic regions showed that average methylation of CpGs within promoter (blood *r* = 0.92, buccal *r* = 0.91, and saliva *r* = 0.94) and genic regions (blood *r* = 0.85, buccal *r* = 0.84, and saliva *r* = 0.89) had higher correlations than those within intergenic regions (blood *r* = 0.78, buccal *r* = 0.74, and saliva *r* = 0.83; Fig. 4). For each genomic region, the highest correlation among the peripheral tissues was the saliva–brain comparison. The variable CpGs from the within-subject correlations were also parsed into their respective genomic regions. The blood–brain correlations were higher in promoter (mean blood *r* = 0.23, buccal *r* = 0.19, and saliva *r* = 0.15) than genic (mean blood *r* = 0.19, buccal *r* = 0.14, and saliva *r* = 0.12) and intergenic regions (mean blood *r* = 0.19, buccal *r* = 0.15, and saliva *r* = 0.13). Of the variable CpGs that are correlated at nominal significance for each peripheral tissue, the greatest proportions are located in genic regions (blood 51.7%, buccal 49.7%, and saliva 51.2%) followed by intergenic (blood 33.3%, buccal 34.1%, and saliva 34.0%), then promoter regions (blood 20.7%, buccal 22.0%, and saliva 20.1%).

Additionally, the level of correlation between DNAm in brain and peripheral tissues based on the functional context of the CpGs was evaluated (Fig. 4). Average methylation of CpGs located within regulatory regions, including transcription factor binding sites, enhancers, and open chromatin, had higher levels of correlation (blood *r* = 0.88, buccal *r* = 0.87, and saliva *r* = 0.91) than those outside of regulatory regions (blood *r* = 0.80, buccal *r* = 0.76, and saliva *r* = 0.84). Among the regulatory features, correlations of CpGs within open chromatin (blood *r* = 0.87, buccal *r* = 0.84, and saliva *r* = 0.90) had higher correlations than CpGs located within enhancers (blood *r* = 0.81, buccal *r* = 0.80, and saliva *r* = 0.86). Similar correlations across individual variable CpGs were seen for CpGs located within regulatory regions (mean blood *r* = 0.19, buccal *r* = 0.15, and saliva *r* = 0.12) as those outside of regulatory features (mean blood *r* = 0.20, buccal *r* = 0.15, and saliva *r* = 0.13), and comparable proportions of variable and significantly correlated CpGs were located within regulatory regions (blood 48.4%, buccal 50.2%, and saliva 48.2%) as those outside them (blood 51.6%, buccal 49.8%, and saliva 51.8%).

### Correlation of psychiatric-associated genes

As a goal of this study is to provide a resource for psychiatric/neurological DNAm studies that rely on peripheral tissues, the peripheral-brain correlations across candidate psychiatric genes, including FK506 binding protein 5 (*FKBP5)*, the glucocorticoid receptor (*NR3C1)*, brain-derived neurotrophic factor (*BDNF)*, the serotonin transporter (*SLC6A4)*, aryl-hydrocarbon receptor repressor (*AHRR*), spindle- and kinetochore-associated complex subunit 2 (*SKA2*), and corticotropin-releasing hormone (*CRH)*, were assessed (Supplemental Figure 5). Variable levels of correlation among the peripheral tissues were revealed for each gene with across-subject correlations for each gene ranging from rho = 0.77–0.99 and within-subject correlations ranging for the individual CpGs of rho = 0.00–0.41, with the highest peripheral correlations varying by gene (Supplemental Table 4).

An important consideration of this study is its potential applicability to the evolving field of psychiatric epigenetics research as new findings of associations emerge for previously unstudied genes. Therefore, a broader picture of the utilization of peripheral tissues for DNAm studies in psychiatric studies was assessed by investigating the brain-peripheral tissue correlations of genes with variants implicated by GWASs of schizophrenia, bipolar disorder, and MDD^31–33^. For this gene set (*N* = 366, see Supplemental Table 5), saliva showed the highest correlation (*r* = 0.91), followed by blood (*r* = 0.88) and then buccal tissue (*r* = 0.87). The correlations for these genes are comparable to the overall level of correlation for genic regions (Fig. 4). Within-subject correlations for these psychiatric disorder-associated genes showed blood (*r* = 0.17; 23% nominally correlated) had the highest mean of the individual variable CpG correlations compared to buccal tissue (*r* = 0.13; 18% nominally correlated) and saliva (*r* = 0.11; 16% nominally correlated). In this gene set, 279 genes had at least one nominally correlated CpG in buccal, and 292 genes had at least one in blood and saliva tissues.

For these analyses, of particular interest are the CpGs in GWAS associated genes that are also variable in brain. The mean of the individual CpG correlations of blood–brain was the highest (*r* = 0.14; 19% nominally correlated), followed by the buccal–brain (*r* = 0.12; 17% nominally correlated) then saliva–brain (*r* = 0.08; 13% nominally correlated). Slightly higher mean correlations are seen for CpGs that are variable in both brain and in the compared peripheral tissue (blood *r* = 0.18; 24% nominally correlated; buccal *r* = 0.13; 19% nominally correlated; saliva *r* = 0.10; 15% nominally correlated).

In addition to genes with GWAS findings, potentially relevant genes include those that are expressed in the brain. Because the majority of genes are brain-expressed, we chose to analyze genes expressed at a medium or high level (6,635 genes). For the across-subject analysis, the saliva–brain correlation was higher than brain correlations with both blood and buccal tissues (blood–brain rho = 0.89, buccal–brain rho = 0.89, saliva–brain rho = 0.92). For the within-subject analysis, blood had the higher mean correlation for the individual variable CpGs (*r* = 0.20; 27% nominally correlated) than did buccal (*r* = 0.14; 19% nominally correlated) or saliva tissues (*r* = 0.11; 16% nominally correlated).

### mQTL analysis

Across-subject comparison of the correlations of DNAm from peripheral tissue to brain showed the non-mQTL CpGs had higher correlations (blood–brain *r* = 0.91, buccal–brain *r* = 0.90, and saliva–brain *r* = 0.94) than the CpGs within mQTLs (blood–brain *r* = 0.78, buccal–brain *r* = 0.81, and saliva–brain *r* = 0.85). For the within-subject analysis, the proportion of variable-correlated CpGs at nominal significance was higher for CpGs within mQTLs (blood 39.4%, buccal 35.4%, and saliva 29.6%) than within non-mQTLs (blood 27.8%, buccal 24.0 %, and saliva 19.0%).

To address how mQTLs may come into play, we additionally analyzed whether regulatory or non-regulatory CpGs may be impacted by their mQTL status. A comparison of the CpGs in mQTLs vs. those in non-mQTLs showed that the proportions were similar for each of these groups, both within regulatory regions (mQTL 35.6%, non-mQTL 35.4%) and outside of them (mQTL 64.4%, non-mQTL 64.6%). Despite this, the variable CpGs in mQTLs showed higher proportions of significantly correlated CpGs in regulatory regions (blood 39.9%, buccal 36.5%, and saliva 29.0%) than did the non-mQTL CpGs in regulatory regions (blood 29.9%, buccal 28.6%, and saliva 20.6%). Similar results were seen for the proportion of variable mQTL CpGs located in non-regulatory regions (blood 39.1%, buccal 34.2%, and saliva 29.8%) than the non-QTL CpGs (blood 27.1%, buccal 22.6%, and saliva 18.6%). Whether comparing all CpGs, CpGs in regulatory regions, or CpGs outside them, the breakdown of correlated CpGs resulted in similar proportions, depending on whether the CpGs were mQTLs or non-mQTLs.

For the previous analysis, which determined the correlation of CpGs in genes with SNPs implicated in GWAS of psychiatric disorders, 7,441 of the CpGs overlapped with the 450K dataset. Of these, the mQTL CPGs showed higher proportions for the variable CpGs that were significantly correlated (blood 29.5%, buccal 28.3%, and saliva 23.0%) than for the non-mQTL CpGs (blood 24.1%, buccal 20.2%, and saliva 16.5%).

### Correlation of FAC-sorted brain samples

Fluorescence-activated cell sorting (FACS) was performed to separate cells positive for a neuronal marker, resulting in five neuronal positive samples and twelve neuronal negative ones with sufficient DNA quantity for analysis. Neuronal negative sorted brain cells had higher correlations to unsorted brain tissue than did neuronal positive samples (neu(−) to unsorted brain tissue *r* = 0.99, neu(+) to unsorted brain tissue *r* = 0.92). This is consistent with the unsorted brain tissue samples having a predominance of glial cells, and also with a previous report that NeuN(−) samples were more strongly correlated than NeuN(+) samples to bulk brain tissue when prefrontal cortex was FAC-sorted and DNAm in promoters across the genome was assessed^34^. In relation to peripheral tissues, neuronal negative samples were more highly correlated than neuronal positive samples for each peripheral tissue (blood to neu(−) *r* = 0.85, blood to neu(+) *r* = 0.79; buccal to neu(−) *r* = 0.84, buccal to neu(+) r = 0.80; saliva to neu(−) *r* = 0.89, saliva to neu(+) *r* = 0.82).

### Correlation of cell-type adjusted samples

Because cell composition estimation and adjustment methods are not available for buccal or saliva tissues, we adjusted the previous findings for cellular heterogeneity strictly using the blood and brain samples in order to compare them. For the across-subject comparison, similar levels of correlation were seen for unadjusted (blood–brain rho = 0.86) and adjusted datasets (blood–brain rho = 0.86). The within-subject analysis of variable CpGs revealed comparable findings for the mean of the correlation values and the proportion of correlated CpGs (unadjusted: mean blood–brain rho = 0.20, 26.6% nominally correlated; adjusted: mean blood–brain rho = 0.21, 28.3% nominally correlated).

### Image-CpG

A website, Iowa Methylation Array Graphing Experiment for Comparison of Peripheral Tissue and Gray matter (IMAGE-CpG), has been created that allows researchers to interrogate DNAm levels and degree of correlation of individual CpGs, genes, or any specified region. It includes data from both the Illumina 450K platform (12 subjects; brain, saliva, and blood tissues) and the Illumina EPIC platform (21 subjects; brain, saliva, blood, and buccal tissues). It can be accessed at https://han-lab.org/methylation/default/imageCpG.

## Discussion

This study provides a resource on the concordance of genome-wide DNAm across three commonly used peripheral tissues with DNAm in live brain tissue from humans. Our data are consistent with the findings of Smith et al.^15^, with the highest correlation to brain seen in saliva rather than blood when the average global correlations are used. As in Walton et al.^17^, Hannon et al.^16^, and Edgar et al.^18^, we observed a limited proportion of peripheral CpGs that were significantly correlated with brain DNAm using a within-subject design. We found a higher proportion of nominally correlated CpGs within our EPIC dataset than those previous studies that compared blood and brain within subjects using the 450K. Importantly, we expanded our analysis to include additional peripheral tissues and found that, in contrast to results obtained using the average global correlations for all CpGs, we now found that blood appeared to show the strongest correlation with brain.

Our two different approaches to correlation yielded results that might appear to be markedly different. The across-subjects results show a high degree of similarity in DNAm across tissues, whereas the within-subjects results suggest most CpGs showed dissimilar patterns. The latter tells us that only about ~21% of CpGs show nominal correlation between blood and brain. One might argue that only these ~171,000 CpGs should be the focus of neuropsychiatric investigation. We did observe that among 366 GWAS-identified psychiatric genes, 292 had at least one of these moderately correlated variable CpGs in them. We also note that the modest sample size we employed limited the variability that could be detected, which, in turn, limited our power to see significant correlations.

By estimating the epithelial cell proportion within their saliva samples based on a buccal DNAm public dataset, Smith et al.^15^ reported that the saliva samples with higher epithelial content were more similar to brain with regard to DNAm. They indicated this could be due to the shared ectodermal origin of epithelial and brain cells or because of the increased variability of methylation in saliva samples. To test these two possibilities, we included buccal, saliva, and blood samples in our analysis. If the former were true, then buccal samples would have the highest correlation among the peripheral tissues. However, this was not supported, and it appears instead that it is the variability of cell types within saliva that contributes to its increased correlations with brain. The MDS plot (Fig. 1) clustered saliva between buccal and blood samples, and it is possible the mixed proportion of the different cell types within saliva enables saliva DNAm to more closely align with the DNAm patterns of the brain. In support of this speculation, the higher the correlation between buccal tissue and blood in our samples, the higher were the correlation of buccal tissue and brain (Supplemental Figure 3), supporting the idea that mixed cell proportions contribute to the DNAm concordance to brain.

There are limitations to this study. First, the infrequent occurrence of neurosurgical cases limits the size of the cohort; however, this is the first study to use direct comparison of live human brain tissues to three commonly used peripheral tissues. Second, each tissue in this study consists of heterogeneous cellular populations, and epigenetic patterns can be cell-type specific. We made limited adjustments for this, although we were constrained by the absence of available methods to apply to buccal and saliva tissues. Although we would have liked to further explore the degree of correlation between peripheral tissues and the FAC-sorted brain tissue, the small sample size (NeuN(+) = 5) limited additional analyses. Third, the cohort in this study consists of patients with epilepsy, which could limit the generalizability of the findings. Fourth, we recognize the wide age range in our study may constrain our ability to detect correlated CpGs. We made use of this cohort as it allowed us to evaluate live human brain tissue, and thus to bypass the confounding factors that are present with postmortem tissue.

This study extends previous work that has investigated the correspondence of DNAm between peripheral tissues and the brain^13–18^. Importantly, although it utilizes live brain tissue samples taken in close timing to three different peripheral tissues within the same individuals. Our data constitute a resource for researchers using peripheral tissues for epigenetic studies of psychiatric/neurological phenotypes. To assist researchers in determining which peripheral tissue is most appropriate for a given study or how findings in peripheral tissues can be understood in the context of the brain, a website for the interrogation of the DNAm concordance across individual CpGs and genes within the Methylation450K/EPIC arrays has been created.

## Supplementary information

Supplementary File Legends

Supplemental Figure 1

Supplemental Figure 2

Supplemental Figure 3

Supplemental Figure 4

Supplemental Figure 5

Supplemental Table 1

Supplemental Table 2

Supplemental Table 3

Supplemental Table 4

Supplemental Table 5

## Data Availability

The DNA methylation microarray data that support the results of this study have been deposited in the Gene Expression Omnibus database (GEO) with the primary accession code GSE111165 (https://www.ncbi.nlm.nih.gov/geo/query/acc.cgi?acc=GSE111165).
