# Supplementary material for: Genome-wide DNA methylation comparison between live human brain and peripheral tissues within individuals
Source: Transl Psychiatry. 2019 Jan 31;9:47. doi: 10.1038/s41398-019-0376-y (PMC6355837; doi:10.1038/s41398-019-0376-y)
Supplement: Supplementary file 1 — Supplementary File Legends [file 41398_2019_376_MOESM1_ESM.docx]

**Supplementary Information**

**Supplemental Tables**

**Supplemental Table 1.** Subject and brain sample characteristics from both the 450K and EPIC datasets. Subjects 125 and 126 were removed from downstream analyses due to pathological reports of oligodendroglioma. Day 0 is indicative of samples taken the day of surgery; those taken before surgery the day of are labeled “’pre”). FCD: focal cortical dysplasia, Tcx: temporal cortex, Amy: amygdala, Hip: hippocampus.

**Supplemental Table 2.** Nested primers used for PCR and sequencing primers for pyrosequencing. Inner primers biotinylated at the 5-prime end are denoted with /5Biosg/ at the start of the sequence. Outer primers were designed using Primer 3 (http://bioinfo.ut.ee/primer3-0.4.0/) while inner and sequencing primers were designed using PyroMark Assay Design software version 2.0 (Qiagen).

**Supplemental Table 3**. Characteristics of the CpGs that surpassed the Bonferroni level of significance for the degree of correlation between brain and peripheral tissues.

**Supplemental Table 4**. DNA methylation correlation between brain and peripheral tissues in CpGs of candidate genes.

**Supplemental Table 5.** Psychiatric associated genes manually assembled from GWAS findings of major depressive disorder, schizophrenia, and bipolar disorder. Abbreviations. SCZ: schizophrenia, BP: bipolar disorder, MDD: major depressive disorder.

**Supplemental Figures**

**Supplemental Figure 1.** Separation of NeuN + and NeuN- nuclei from human brain tissue by fluorescence-activated cell sorting and validation by bisulfite pyrosequencing. (A, B) Representative gating strategy performed on all samples using (A) forward scatter area vs. side scatter area and (B) forward scatter area vs. forward scatter width. (C-F) Gating by PerCP-Cy5.5 and Alexa fluor 488 signal. NeuN- nuclei were identified using Alexa Fluor® 488 signal from isotype control antibody and PerCP-Cy5.5 autofluorescence. In contrast, NeuN+ nuclei were identified with increased Alexa Fluor® 488 signal and diminished PerCP-Cy5.5 autofluorescence. Staining of nuclei using isotype antibody for (C) patient A and (D) patient B. Staining of nuclei using anti-NeuN antibody for (E) patient A and (F) patient B. (G) Bisulfite-pyrosequencing at the GFAP promoter. DNA was extracted from NeuN+ and NeuN- nuclei from patient A and patient B (N = 2) and bisulfite converted prior to bisulfite-pyrosequencing. Figure shown is mean ± S.E.M.

**Supplemental Figure 2.** Multidimensional scaling plot from genome-wide DNAm of preliminary analysis of tissue samples analyzed on the Illumina 450K array. The two brain outliers are samples consistent with oligodendroglioma and were removed from downstream analyses.

Supplemental Figure 3. Comparison of the buccal-brain correlation with the correlations of buccal-blood and buccal-saliva. A The degree of association between the buccal-brain correlation to that between buccal-blood (r^2^ = 0.88, *F* = 142.8, p = 2.8 x 10^-10^). B The degree of association between the buccal-brain correlation to that between buccal-saliva (r^2^ = 0.01, *F* = 0.21, p = 0.65).

**Supplemental Figure 4.** The calculated distance from the MDS plots between each peripheral tissue and its corresponding brain sample is plotted against the level of correlation among those samples.

Supplemental Figure 5. Average DNA methylation (β) patterns in candidate psychiatric genes across blood, saliva, buccal, and brain tissues. A FK506 binding protein 5 (*FKBP5)*, B the glucocorticoid receptor (*NR3C1)*, C brain-derived neurotrophic factor (*BDNF)*, D the serotonin transporter (*SLC6A4)*, E aryl-hydrocarbon receptor repressor (*AHRR*), F spindle and kinetochore associated complex subunit 2 (*SKA2*), and G corticotropin-releasing hormone (*CHR)*.
