# Supplementary figures and images for: Genome-wide DNA methylation comparison between live human brain and peripheral tissues within individuals
Source: Transl Psychiatry. 2019 Jan 31;9:47. doi: 10.1038/s41398-019-0376-y (PMC6355837; doi:10.1038/s41398-019-0376-y)

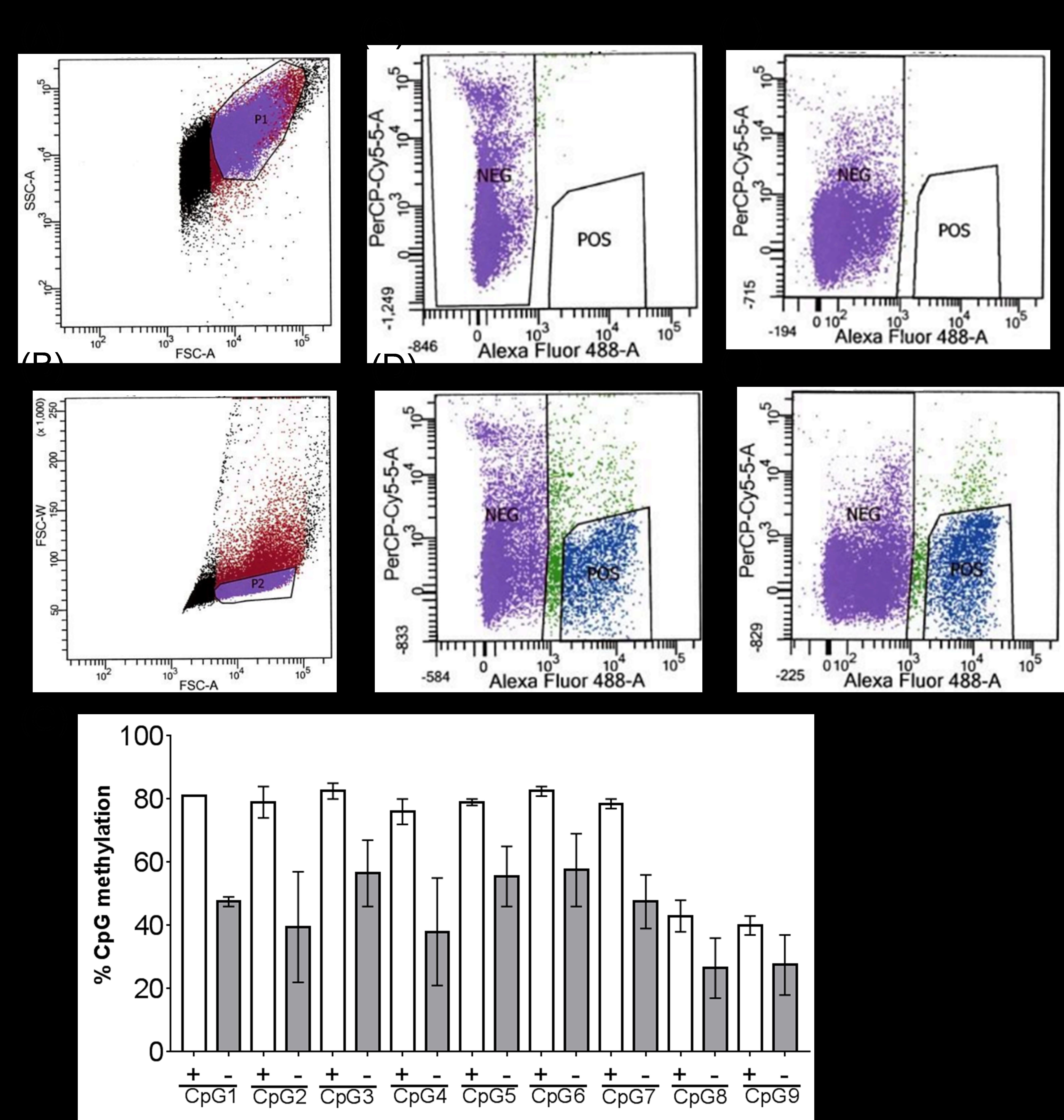

Supplement: Supplementary file 2 — Supplemental Figure 1 [file 41398_2019_376_MOESM2_ESM.jpg]

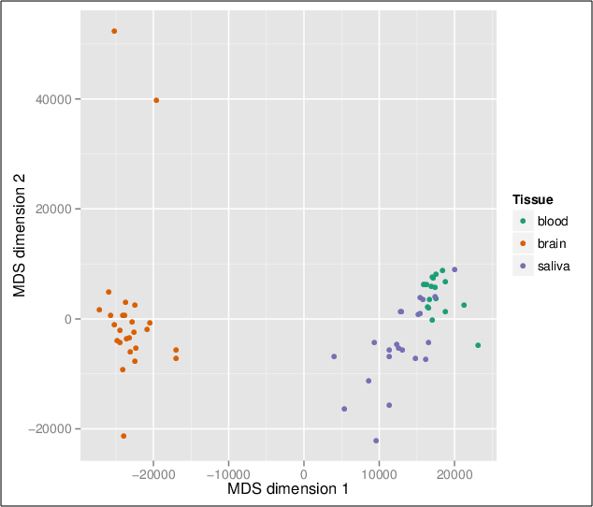

Supplement: Supplementary file 3 — Supplemental Figure 2 [file 41398_2019_376_MOESM3_ESM.jpg]

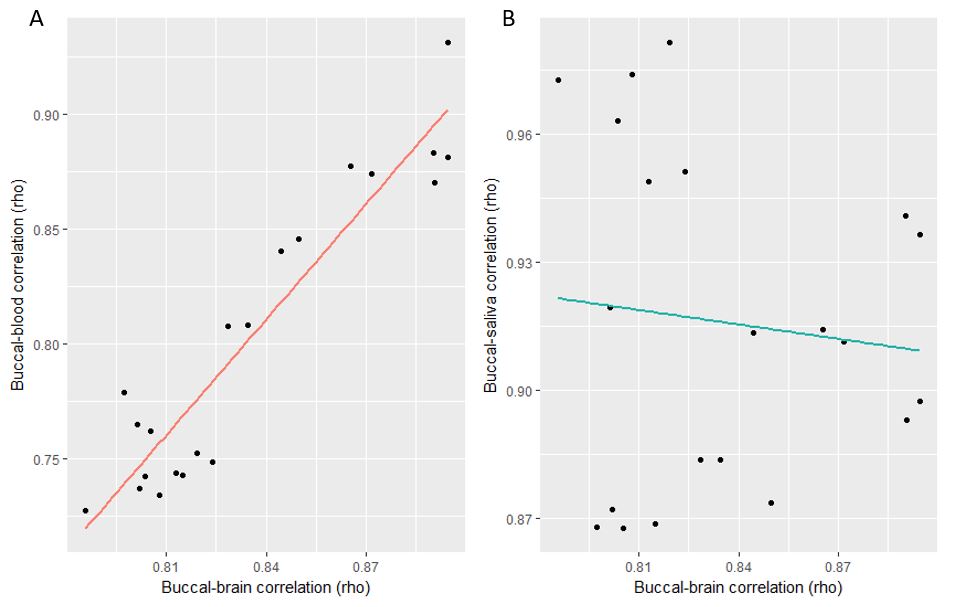

Supplement: Supplementary file 4 — Supplemental Figure 3 [file 41398_2019_376_MOESM4_ESM.jpg]

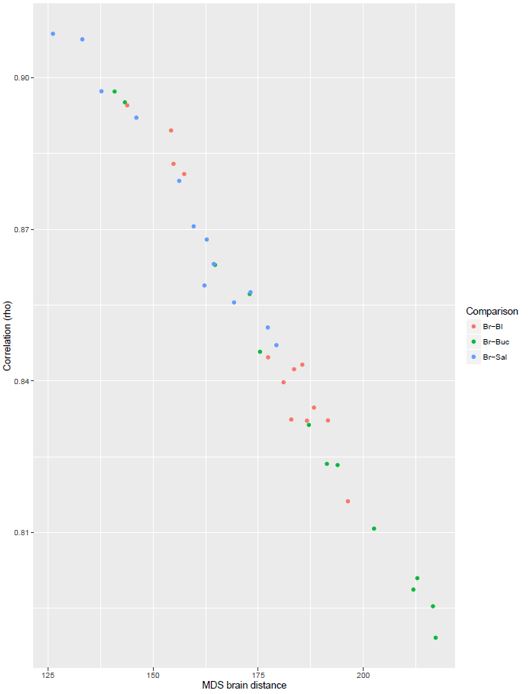

Supplement: Supplementary file 5 — Supplemental Figure 4 [file 41398_2019_376_MOESM5_ESM.jpg]

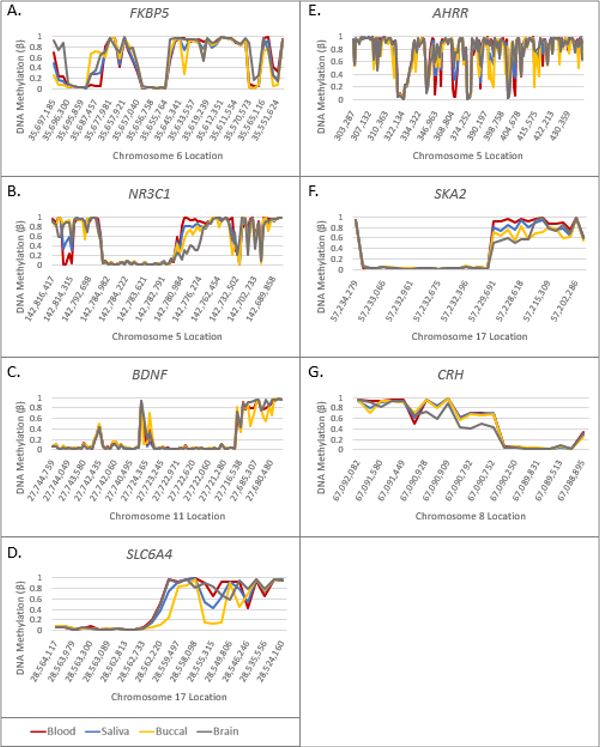

Supplement: Supplementary file 6 — Supplemental Figure 5 [file 41398_2019_376_MOESM6_ESM.jpg]
